# First Systematic Review on Health Communication Using the CiteSpace Software in China: Exploring Its Research Hotspots and Frontiers

**DOI:** 10.3390/ijerph182413008

**Published:** 2021-12-09

**Authors:** Qiong Dang, Zhongming Luo, Chuhao Ouyang, Lin Wang

**Affiliations:** Climate and Health Communication Research Center, School of Journalism and Communication, Guangxi University, Nanning 530004, China; zhongminggin@163.com (Z.L.); 18779666331@163.com (C.O.); leenw@foxmail.com (L.W.)

**Keywords:** health communication, CiteSpace, research hotspots, development process, research frontier, China

## Abstract

Since the 1970s, health communication (HC) has attracted widespread attention from practitioners and researchers in various fields in China, leading to the production of a vast array of literature. In order to reveal the current state, popular themes, and research frontiers of HC research, this study employed the CiteSpace software to conduct a comprehensive review based on 1505 HC publications from 1992 to 2021 retrieved from the China National Knowledge Infrastructure (CNKI) database. The results demonstrated that (1) the number of HC publications has experienced an annual increase over the past 20 years, albeit with certain inverted S-shaped fluctuations and (2) the most prolific authors mainly included Wang L.Y., Zhang Z.L., and Wang Y.L., while well-known universities played a leading role in HC research in China. A significant finding was that a stable core group of authors or institutional has been not formed in the HC field. Furthermore, (3) research hotspots included health education, new media, health literacy, health information, animal husbandry and veterinary medicine (AHVM), the doctor–patient relationship, and public health emergencies. Additionally, the development of the field could be divided into four stages, indicating a significant shift in HC research from focusing on medicine and public health issues towards communication issues. Finally, (4) new research frontiers have mainly included the WeChat official account and Health China.

## 1. Introduction

The outbreak of coronavirus disease (COVID-19) prompted the academic community to reconsider HC, and as a result it has become one of the most important research topics worldwide in the past two years. Essentially, HC first arose as a significant issue in the public health field in the United States in the 1970s. More specifically, the Stanford Heart Disease Prevention Program conducted in 1971 was considered to mark the very beginning of HC research worldwide. By the 1980s, research on HC would gradually become a significant concern, spreading from the United States to other countries/regions, including Europe, Asia, Australia, and China [1,2,3,4].

At the beginning, a significant body of international scholars such as Thomas [5], Thompson et al. [6], Wright et al. [7], and Schiavo [8] explored the concept and connotations of HC. In fact, the concept of HC was formally introduced by Jackson [9] for the first time in 1992, while the most widely accepted definition has been proposed by Rogers [10] in 1994. Moreover, the majority of HC definitions have stressed that the ultimate goal of HC is to ameliorate the public health index and encourage healthy behavior, with the behavioral change models widely used in HC research mainly including the health belief model, the social cognitive model, and the theory of reasoned action [11]. Subsequently, a range of HC studies have been conducted from the perspectives of medicine and public health, social marketing, communication, and pedagogy [1,12,13,14]. Clearly, HC is a complex research issue that is deeply connected to a variety of different disciplines. In addition, the analysis of previous studies illustrated that HC research worldwide has focused on several themes, mainly including the doctor–patient relationship [12], HIV prevention and control [15,16], media interferences [13,14], the effect of HC [17], the environment of HC [18], health promotion, health education and health literacy [19,20,21], and special topics (e.g., euthanasia [22] and homosexuality [23]).

In general, the above-mentioned studies laid a solid foundation for follow-up HC research, especially in providing a reference for China’s HC research. On the global scale, China has the world’s largest population; therefore, HC research there has become extremely significant, especially during public health emergencies. China’s HC research can be traced back to the late 1980s, with health education then regarded as forming the basis for HC research [4]. Subsequently, the concept of HC in a complete sense was introduced into China via a cooperative public health project between the United Nations Children’s Fund and the Chinese Government (1989–1993).

Since then, many research achievements in HC have emerged in China. For example, previous HC reviews mainly focused on certain specific subfields and themes. To illustrate, Hong et al. [24] reviewed 28 articles to examine the intervention measures applicable to female sex workers for the prevention of HIV in China. Along different lines, following an analysis of 75 empirical studies published over the previous 15 years, Tang et al. [25] found that social media formed the main channel for the public to access and acquire knowledge on homosexuality in China. Furthermore, a review conducted by Hu and Wang [26] found, through an analysis of 58 studies, that traditional Chinese sports communication can reap further health benefits for older adults in China. Such reviews can deepen the overall understanding of specific aspects of HC; however, they do not provide readers with an overall understanding of HC research in China. Additionally, according to Hosseini et al. [27] and Darko et al. [28], qualitative reviews can only analyze a limited number of documents and might result in a potential bias through subjectivity. Therefore, it is necessary to employ the quantitative method in systematically reviewing the wide array of HC literature in China.

Therefore, we conducted a comprehensive bibliometric analysis using the CiteSpace software to review HC research from 1992 to 2021. To the best of our knowledge, this is the first study to have applied bibliometric visualization tools to a systematic investigation of the active authors, journals, institutions, and keywords involved in China’s HC research, thus highlighting the research hotspots and tracking emerging trends in order to offer references for further research. We considered that this study could not only help researchers to extract potentially valuable information for deeper investigation but also provide them with meaningful guidance in the selection of frontier topics. This study aims to address the following three key research questions (RQ)in the context of China.

RQ1. What is the chronological growth of published articles on HC research in China?

RQ2. Who are the major contributors (authors and institutions) in HC research in China?

RQ3.What are the research hotspots and research frontiers in HC research in China?

Research hotspots means the significant research topics/themes in a specific field and in a certain period [29], while research frontier refers to the state-of-the-art thinking in a research field in a certain period [30]. The remainder of the current study is arranged as follows. Section 2 introduces the materials and research methods, including the data collection procedure, the bibliometric analysis, the analysis tool, and the paths of the analysis. Section 3 discusses the temporal distribution of publications on HC research in China. Section 4 identifies the major contributors to HC research in China through a co-authorship analysis, including influential authors and core institutions, while Section 5 identifies the research hotspots and their development over time, together with the research frontiers, through a co-word analysis. Finally, Section 6 summarizes the key conclusions of the current study and points out limitations and future research directions.

## 2. Materials and Methods

### 2.1. Data Collection Procedure

**Figure 1:**
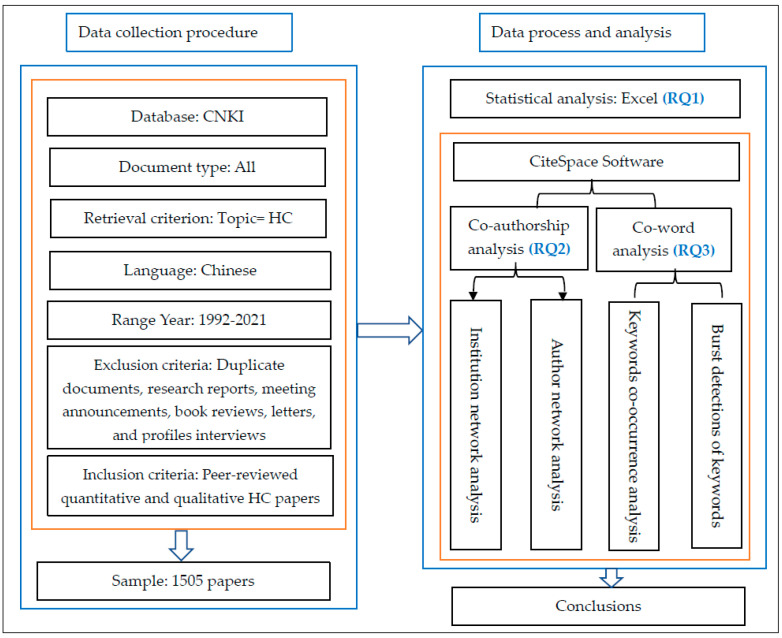
The integrated analysis framework.

The data collection procedure for the current study is presented in Figure 1. First, we collected the data from CNKI, which is the largest and most authoritative database platform for scientific literature in China. Moreover, CNKI has a collection of scientific literature from more than 7000 academic journals, which ensures the representativeness and authority of the literature sources. Secondly, we used the term health communication as the topic, in order to retrieve all of the relevant documents published from 1992 to 31 August 2021, generating a total of 1665 documents. The major reason why this study selected HC as the research topic is because since the outbreak of COVID-19 in 2020 in China HC has attracted great attention from the public and the Chinese Government [31], driving scholars to reconsider HC research in China. Moreover, to the best of our knowledge, there is currently no study that has conducted a quantitative review of HC using CiteSpace in China. We set 1992 as the beginning of our date range because the first relevant article appeared in the CNKI database in 1992. In addition, the articles in the CNKI database were written in Chinese. Thirdly, among all the various types of documents available, articles published in journals were found to be relatively more continuous, sensitive, and directly related to the academic field [29]. In order to ensure the rationality and validity of the data, we carefully read the 1665 documents selected and manually removed a total of 160 documents of various types, such as duplicate documents, research reports, meeting announcements, and book reviews. Only peer-reviewed, published, quantitative and qualitative journal articles were selected as the targets of this research. Finally, a total of 1505 articles were selected, and subsequently, these 1505 selected publications concerning HC were processed in CiteSpace on 1 September 2021. The full sample of papers is available on request.

### 2.2. Bibliometric Analysis

Bibliometrics refers to the application of mathematical and statistical methods to objectively analyze the nature of the spatial distribution of scientific literature in a certain period in a specific field [32,33]. Bibliometrics is centered on three empirical statistical laws: (1) Lotka’s law, put forward in 1926, reveals the distribution of the authors of scientific literature [34]; (2) Zipf’s Law, proposed in 1948, identifies the distribution of word frequencies in the literature [35]; (3) Bradford’s Law, put forward in 1934, characterizes the distribution of publications in journals in a certain discipline [36]. Each bibliographic record contains four textual fields: title, abstract, author keywords, and keywords plus. Bibliometrics analysis covers three main analyses: co-authorship analysis, co-word analysis, and co-citation analysis. These analyses can be used to reveal the collaborative relationships of authors, institutions, and countries, and to identify influential researchers, research hotspots, research frontiers, and future research trends in certain fields, and therefore to reveal the social structure, intellectual base, and knowledge structure of a specific field [30,32].

In our study, we mainly conducted a co-authorship analysis and a co-word analysis. (1) Co-authorship is a major type of scientific collaboration, and since the 1960s scholars have displayed great interest in utilizing co-authorship to measure research collaborations at authorial, institutional, and international levels [37]. Subsequently, social network analysis was introduced in co-authorship analysis to better understand the various types of research cooperation [38,39]. Social network analysis is a quantitative research method that focuses on the relationships between actors in social networks and characterizes the structure of a network based on nodes (e.g., authors, institutions, and countries) and the connections or links (relationships) between them [38]. Moreover, in co-authorship networks, two authors who co-authored a research paper together are connected [37]. (2) Co-word analysis is a content analysis method that involves statistically analyzing the co-occurrence of subject words in a field of literature [40]. Moreover, this method has been used to identify important research themes within a particular research area, as well as the development of these research themes over time, research frontiers, and emerging research trends, by analyzing the frequencies of words present in the literature [40,41]. Additionally, co-word analysis typically includes keyword co-occurrence analysis and burst keyword analysis, with the former being used to identify research hotspots, while the latter is employed to explore research frontiers [41].

To date, bibliometric analysis has been used in a wide array of fields, such as environmental science, forestry, management, tourism, and agriculture. For example, Chen [42] conducted a bibliometric analysis using the CiteSpace software with the aim of revealing the knowledge structure of the research concerning mobile technology in tourism.

### 2.3. Analysis Tools and Paths of Analysis

#### 2.3.1. CiteSpace Software

CiteSpace software, as a knowledge management tool, can be used to conduct bibliometric analysis. It is run in a Java environment and was first created by Chen MeiChao, a professor working in the College of Information Science and Technology at Drexel University in the United States [41]. To date, the CiteSpace software has been used in various fields such as environmental science, pedagogy, forestry, communication, public health, and tourism [43,44]. Moreover, a significant body of scholars worldwide have utilized it to handle mass corpuses of literature in order to track research hotspots, research frontiers, and emerging research trends. For example, Goerlandt et al. [45] employed CiteSpace to conduct a co-citation analysis to reveal the knowledge base in the field of risk communication, while Zhao. et al. [46] used CiteSpace to identify popular research issues and future research directions in the field of human health and climate change.

In this study, we used CiteSpace V.5.8.R2 to conduct a bibliometric analysis based on two considerations. First, it can be easily accessed for free, and it is capable of objectively processing large-scale literature data, as well as providing a visualization map. Secondly, we tested other bibliometric analysis tools such as VOSviewer, BibExcel, and UCINET and found that CiteSpace can better recognize Chinese characters, thus avoiding error (or unintelligible) codes, and therefore ensuring the reliability of the results. Moreover, it stands out among other visualization tools such as VOSviewer and Gephi because it offers the most comprehensive suite of tools for generating multiple bibliometric networks and conducting multiple methods of analysis [41,47]. Different types of bibliometric networks can be constructed in CiteSpace, mainly including co-authorship analysis, keyword-occurrence analysis, and co-citation analysis.

The general procedures for visualization analysis using CiteSpace include six main steps (see Li and Chen [41] and Chen [48]): (1) install the CiteSpace software in a Java environment; (2) collect and download data; (3) set parameters; (4) run CiteSpace; (5) run data visualization; (6) export visualized maps. The parameters in CiteSpace should be appropriately selected according to the research objectives. For more information on how to operate CiteSpace software to conduct bibliometric reviews in specific areas, please refer to the literature (see Li and Chen [41] and Chen [48,49]).

#### 2.3.2. Two Paths of Analysis

Before running the CiteSpace software, the parameters were set as follows: (1) a time span was chosen ranging from 1992 to 2021; (2) the year per slice was set as 1; (3) the node type was set as author/institution/keyword; (4) threshold selection criteria were set for the top 25, which means that data were extracted on the top 25 results for each time slice; (5) pruning was set as none/MST. The remaining parameters were the default settings. In order to answer the research questions, we used CiteSpace to conduct two paths of analysis: co-authorship analysis and co-word analysis.

(1) Co-authorship analysis. This establishes authors and institutions as nodes in the analysis of bibliometric records, in order to obtain the corresponding cooperation network map. According to the analysis of the collaborative relationship maps, we can identify the collaborative relationships among the authors and institutions and identify influential/productive/core authors and institutions in a specific domain [41,50]. Co-authorship network analysis has been used to assess collaborative relationships and identify influential scholars and institutions [50]. Each collaborative relationship is identified by its density, with density being a measure of the connectivity within a network, defined as the ratio of the number of existing links to the maximum possible number of links in a given network. The value of the density ranges between 1 and 0; if the value of the density is closer to 1 this represents a more cohesive networking relationship and if the value is closer to 0 it indicates a weak cooperative relationship [50]. Lastly, the influential scholars or institutions are identified via frequency (i.e., the number of papers published), with the authors or institutions with a high frequency identified as influential/leading/core authors or institutions. In addition, a co-authorship network map is formed if two authors (nodes) have co-authored an article together (links) [41]. The co-authorship analysis was used to answer RQ2.

(2) Co-word analysis. The analysis of word frequency is a core path of analysis in CiteSpace that is used to identify important research topics and research frontiers [30]. This study took keywords as nodes in the co-word analysis. This is because keywords provide information about the core content of articles. Furthermore, keyword co-occurrence analysis was conducted to generate a keyword co-occurrence network map and a keyword time-zone map, with the significance of the keywords measured using two metrics: frequency and betweenness centrality (BC) [40]. Keywords are words that express the subjective concepts of a book or other form of literature. They are the result of a high level of generalization of the author’s academic thoughts and views and can reflect the core content of the literature under analysis [29]. Therefore, if a keyword repeatedly appears in the literature of a certain field, it can reflect the fact that the particular research topic represented by the keyword should be considered a research hotspot in that field. These research hotspots in a specific field and period of time can be determined by identifying keywords with high frequency. BC is a measure of a node, and is used to measure the probability of any shortest path passing through the node in the network [41]. It shows the contribution of the node to establishing connections with other nodes in the network. Keywords with a higher centrality are more significant and more influential in certain research domains [41]. In addition, if the value of BC is greater than 0.1, it indicates that the keyword has a connection to other keywords throughout the entire network map [51]. A burst keyword analysis was also conducted using CiteSpace. The burst keyword analysis method reveals keywords that have changed rapidly in a short period of time or have dramatically increased in number, emphasizing abrupt changes in keywords [52]. Keywords with a high strength value could be identified as indicating research frontiers. Ultimately, co-word analysis can be used to answer RQ3. The results of the research are presented in Section 3, Section 4 and Section 5 and discussed in combination with the representative literature on HC research in China.

## 3. The Temporal Distribution of Publications on HC Research in China

**Figure 2:**
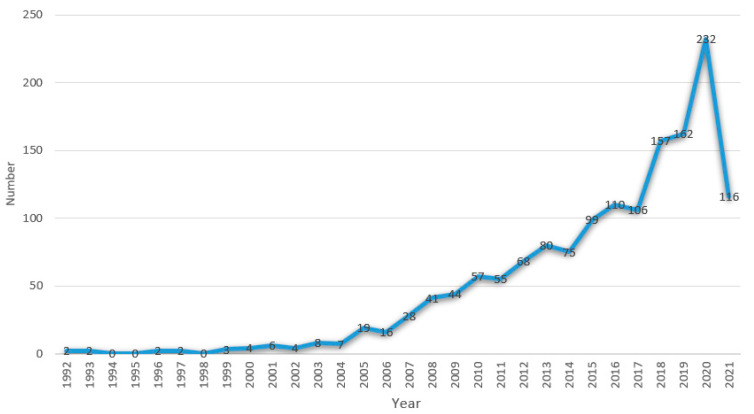
The annual number of publications on HC research in China from 1992 to 2021.

A total of 1505 published documents were found in the selected databases from 1992 to 2021, with the annual number of publications found to have been increasing over the past 20 years, albeit with certain inverted S-shape fluctuations, as can be seen in Figure 2. Furthermore, the lowest outputs appear to have occurred in 1994, 1995, and 1998, which all saw zero publications, while the output reached a peak in 2020 with 232 publications, following nearly three decades of development. The annual average number of papers published was about 75. It is interesting to note that the number of articles published over the past six years was greater than the cumulative number of articles published between 1992 and 2015. Moreover, it is clear that this is still a young field, as most papers were published in the past decade or so.

Three developmental stages can be identified from Figure 2 a slow development stage (1992–2004), a steady development stage (2005–2015), and a rapid development stage (2016–2021).

Slow development stage (1992–2004). During this stage, a total of 40 documents were published, which represents an exceedingly small proportion (2.67%) of the total amount. Significantly, no research records appeared in 1994, 1995, and 1998, indicating that no attention was paid to HC research or that research in this subject still remained at the exploratory stage.

Steady development stage (2005–2015). In this stage, the number of publications increased year on year. During this period, a total of 582 articles were published, accounting for about 39% of the total.

Rapid development stage (2016–2021). During the rapid development stage (2016–2021), 883 papers were published, indicating that HC had become a mainstream research topic since 2016. In particular, the peak occurred in 2020 with 232 articles. A reasonable explanation for this is that the COVID-19 crisis stimulated scholars to reconsider HC in China. Meanwhile, the significance of HC was increasingly recognized by the Chinese Government.

Table 1 lists the top 10 journals which paid the greatest attention to HC in China, although it should be noted that these top 10 journals published only 130 out of the 1505 articles, thus accounting for only a small proportion (8.64%) of all selected articles. Furthermore, this topic attracted the most attention from scholars dealing with communications, public health, and sociology. Among these, communication scholars have a core position in the field of HC. Specifically, the *Chinese Journal of Journalism and Communication* ranked in first place with 30 publications, followed by *Modern Communication* (Journal of Communication, University of China) and *Journalism and Communication*. Significantly, the top three journals are the most authoritative journals and have published the highest-quality papers in the field of communication in China. In addition, journals in the medical and public health fields also contributed significantly—for example, *Chinese Medical Ethics* came fifth with 11 articles. In summary, this research topic is multi-disciplinary and attractive to scholars in various research areas in China.

## 4. Major Contributors to HC Research in China: Co-Authorship Analysis

At present, HC has become a matter of concern, resulting in it receiving varying degrees of attention from scholars and institutions alike. Whether authors and institutions have established a close cooperative relationship is an urgent problem to be investigated in the field of HC. In order to explore the cooperative relationships among authors or institutions and identify the leading authors or institutions in HC research, we conducted a co-authorship analysis using CiteSpace, generating an author cooperation network map and an institution cooperation network map.

### 4.1. Influential Authors: Author Cooperation Network Analysis

**Figure 3:**
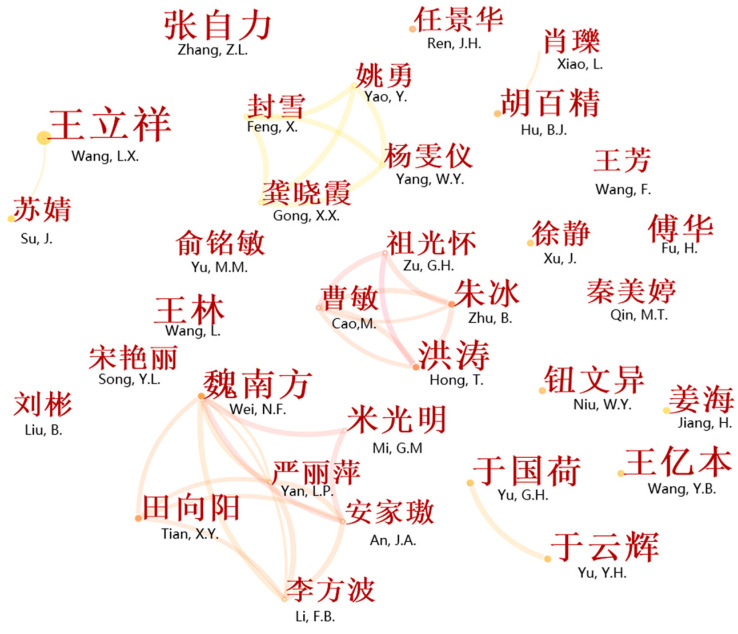
Author’s collaboration network map for the field of HC in China. (Note: the authors added English to the map to aid understanding).

The author cooperation network map was used to investigate the cooperation between authors and identify leading authors in HC research. The current study used CiteSpace to conduct a co-authorship analysis (i.e., the node was set as “author”), producing the author collaboration network map shown in Figure 3. It can be seen that Figure 3 is composed of nodes and lines. The nodes represent the authors, while the lines are used to depict the authorship relationships between nodes. Nodes with a large size (determined by the number of publications) are generally confirmed as significant nodes that may have a large influence on the development of a scientific research field [48]. A thicker line between two nodes illustrates a closer relationship.

Figure 3 consists of 518 authors (nodes) and 439 collaborative relationships (connections). The density of the network map was 0.0026, illustrating that only 0.26% of the potential relationships in the HC network have been realized; this also reveals that strong partnerships among authors have not yet been formed in the HC field. However, according to Figure 3, actualized partnerships among the authors can be identified, including three important collaborative groups. The first group includes Wei N.F, Yan L.P., Mi G.M., An J.A., Li F.B., and Tian X.Y. Apart from Mi G.M. from Hebei University, these authors are all from the China Health Education Center. Their research interests include evaluating the effectiveness of HC and health care promotion between children and mothers [53]. The second group consists of Cao M., Hong T., Zhu B., and Zu G.H, who are all from the Anhui Center for Disease Control and Prevention. They focus on research concerning public health emergency management [54]. The last group consists of Feng X., Yao Y., Yang W.Y., and Gong X.X., who all work in Wuxi People’s Hospital. These authors have demonstrated great interest in the study of advanced technologies, such as artificial intelligence, big data, and virtual reality, especially the applications of these technologies in HC practices [55]. Another interesting finding is that cooperative relationships usually occur between colleagues, which indicates that a national academic community concerned with HC has not yet formed in China.

High-yield authors play a leading role in a research domain and are considered to be the core driving force of the research in their field [41]. In order to identify the core author group, we used Price’s law, i.e., M = 0.749(N Max)/2 [56].

This law is normally used to identify the relationships between the literature items on a particular subject and the number of authors in that subject field. If half of the papers on the same subject matter are written by one group of highly productive authors, and the number of authors in this set is approximately equal to the square root of the total number of authors, then in this formula N Max refers to the number of papers published by the author who published the largest number of papers [56].

In the current study, N max was 9 (see Table 2). Following the calculation, it was found that the most prolific authors in the HC field were those who had published more than three papers. Furthermore, according to the results yielded by CiteSpace, 33 authors had published more than three papers, giving a total of 158 papers (accounting for 10.50% of the total amount). Price posited that it is reasonable to expect that 50% of all papers on the same topic will be written by the most productive authors. However, there is obviously a huge gap between 10.5% and 50%, which indicates that a stable core author group has not yet been formed in the HC field in China.

Table 2 provides a general description of the authors who published five or more papers in the field of HC between 1992 and 2021. These authors can be identified as influential authors in HC research. Wang L.Y. published nine papers and therefore ranked first, accounting for 0.6%, while seven people tied for second place with six publications each. There is little difference in the number of papers published by the top three authors.

### 4.2. Influential Institution: Institution Cooperation Network Analysis

**Figure 4:**
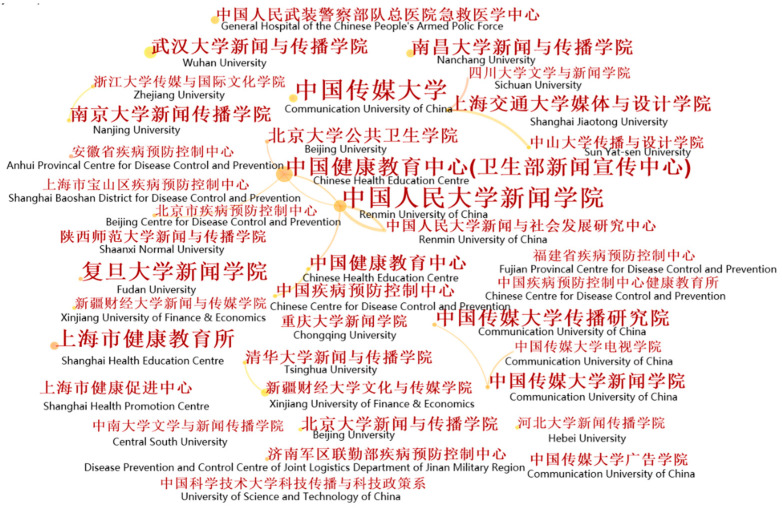
Institutional collaboration network map for the field of HC in China. (Note: the authors added English to the map to aid understanding).

In order to explore the cooperative relationships among institutions and identify influential institutions, we used CiteSpace to generate an institutional network map. Apart from changing the node type from “author” to “institution”, the parameters remained the same. This yielded a network comprising 499 institutions (nodes) and 124 cooperative relationships (lines), as shown in Figure 4. Figure 4 consists of nodes and lines where the nodes represent the institutions, while the lines are used to depict the authorial relationships between nodes. Nodes with a high frequency (regarding the number of publications) are generally identified as significant nodes which may have a large influence on the development of a scientific research field. A thicker line between two nodes illustrates a closer relationship.

The density of the network map was 0.0014, illustrating that only 0.14% of the potential relationships in the HC network had been realized. This also illustrates the fact that collaboration between institutions is weak. Nevertheless, two significant cooperative relationships could be identified from Figure 4. First, the Chinese Health Education Centre had cooperated with Renmin University, Peking University, and Beijing Centre for Disease control and Prevention. Together, they focus on research concerning the relationships among medicine, humanity, the media, and major public emergencies. Shanghai Jiao Tong University established a partnership with Sichuan University and Sun Yat-sen University. Their main research interests include medical science popularization and medical assistance. An interesting finding is that collaboration among institutions generally occurred between different colleges of the same university.

Research institutions with high frequency are identified as influential institutions. The top 10 research institutions shown in Table 3 are ranked according to frequency (i.e., the number of publications) in the field of HC between 1992 and 2021, with a total of 287 articles having been published, accounting for 19.1% of all the selected articles. These institutions are identified as the most influential research institutions and their research represents the latest research directions and the core issues in the HC field in China. Significantly, there are seven universities among the ten research institutions, indicating that universities are the leading force in HC research in China.

The top five research institutions included the Communication University of China with 80 publications, Renmin University (39), Fudan University (39), China Health Education Center (28), and Peking University (26). Another significant finding is that four of the top five universities are well-known universities in China.

## 5. Research Hotspots and Research Frontiers in HC Research in China: Co-Word Analysis

### 5.1. Research Hotspots and Their Development: Keyword Co-Occurrence Analysis

In order to identify the research hotspots and their evolution over time in the HC field, we conducted a keyword co-occurrence analysis using CiteSpace, thereby generating a keyword co-occurrence network map alongside a time-zone network map of keywords. The maps consist of lines and nodes, with the larger-sized nodes indicating that a particular keyword co-occurred more times with other keywords. The thickness of a line further reflects the intensity of the co-occurrence of two keywords [52].

#### 5.1.1. Research Hotspots in the Field of HC in China

**Figure 5:**
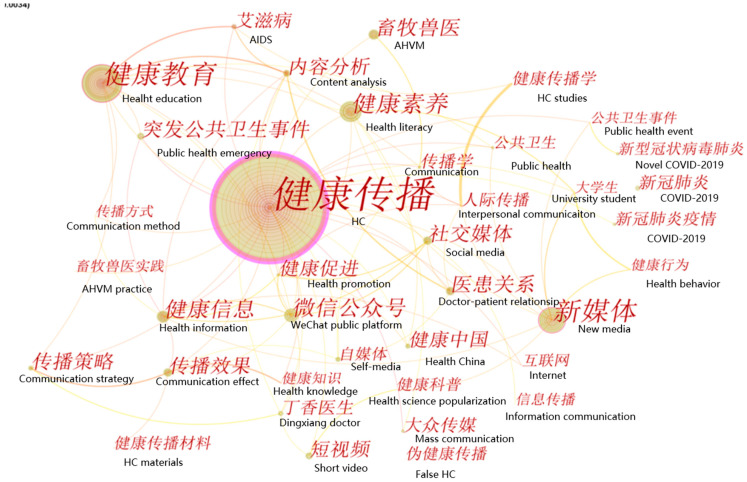
The keyword co-occurrence map for the field of HC from 1992 to 2021 in China. (Note: the authors added English to the map to aid understanding).

In order to identify important research topics in HC research, the study conducted a keyword co-occurrence analysis. The parameters in CiteSpace remained the same, except that the node type was changed from “institution” to “keyword”. The keyword co-occurrence network map shown in Figure 5 consists of 731 institutions (nodes) and 904 connecting lines between nodes. In Figure 5, each node represents one keyword; larger nodes reflect a higher co-occurrence frequency, with the links between each two keywords revealing the co-occurrence relationships.

In accordance with Figure 5, the eleven keywords with the highest frequencies and strongest BCs are presented in Table 4. Keywords with a higher centrality are more significant and hold greater importance in the HC research domain. In the current study, only the BC values of HC, health education, and new media exceeded 0.1, illustrating that these three keywords hold key positions and have a connection with other keywords in the network.

Among the top 11 keywords, HC had the highest frequency, ranking in first place because it was a retrieval term in our study. Therefore, this study did not take it into consideration. In addition, social media and the WeChat public platform were considered to be research branches related to new media. The BC value of new media was found to be higher than those of the WeChat public platform and social media, illustrating that new media played a more significant role in HC research. From the three keywords, we extracted social media as a research hotspot. In addition, COVID-19 formed part of public health emergency. The BC value of public health emergency was found to be higher than that of COVID-19, revealing that public health emergency is more significant in HC research. Therefore, by considering both frequency and BC, we selected public health emergency as the research hotspot. Finally, we identified seven research hotspots as follows: health education, new media, health literacy, health information, AHVM, the doctor–patient relationship and public health emergencies. The following paragraphs will discuss these seven research hotspots in combination with the representative literature in the HC field in China.

Research on Health Education. In China, the primary objective of health education is to educate people in order heighten awareness of health, develop ideal healthy behaviors and lifestyles, and eliminate or reduce risk factors that affect health [57]. In particular, HC is seen as one of the most important means of achieving health education. As such, this research hotspot can be divided into two main research directions. One of these is evaluating the effectiveness of health education. In particular, scholars have paid great attention to evaluating the effectiveness of health education concerning acquired immunodeficiency syndrome/human immunodeficiency virus (AIDS/HIV), infectious diseases such as severe acute respiratory syndrome (SARS), Asian lineage avian influenza A virus (H7N9), pulmonary tuberculosis, adolescent mental illness, and diabetes [58,59,60,61,62]. The subjects of these studies have mainly included teenagers, rural women, army officers and soldiers, and children in poverty-stricken, minority areas. For example, Feng [62] demonstrated that centralized teaching can encourage pregnant women to voluntarily accept HIV counseling and testing. Furthermore, to a large extent, education is recognized as an effective measure for altering the public’s understanding and behavior regarding their health. The other direction is the role of the communication medium in health education. The channels for accessing health education have been broadened in recent years, and there has been a significant body of discussion on the positive role of the Internet, the WeChat public platform, short videos, online games, and blogs communicating about health education [58,59,60,63]. For instance, Jiang et al. [63] found that for the majority of students, awareness regarding health knowledge and healthy practices had been effectively mobilized through their participation in a health education game named “Health Knowledge Mengmeng Answer”.

Research on New Media. Differing from traditional media, new media have changed the way health information is disseminated. This research hotspot has mainly focused on the use of, and changes wrought by, new media in the HC field. Among the types of new media, social media—especially the WeChat public platform—have been considered to be the most popular platforms for disseminating knowledge on health in recent years, especially during the COVID-19 lockdown period in China [64,65]. Using new media to disseminate health information may lead to a series of issues [65]. For example, false health information spreads quickly in the virtual space due to a lack of supervision of the dissemination of health information [66]. In order to deal with this problem, scholars suggested strengthening the position of authoritative experts on HC and building HC channels under the supervision of the government, the public, and enterprises in the Internet space [67,68].

Research on Health Literacy. In China, the study of health literacy first began in 2005, when an article titled “Progress in Health Literacy Research” [69] introduced the concept of health literacy in China for the first time. Following this, scholars began to explore the ways in which health literacy could be evaluated [70,71,72]. They initially drew on evaluation scales from abroad for measuring levels of health literacy, such as the Rapid Estimate Adult Literacy in Medicine and the Test of Functional Health Literacy in Adults scales, to measure China’s health literacy level. Subsequently, in order to develop a health literacy measurement tool in line with the Chinese cultural environment, Xiao [73] established a health literacy evaluation indicator system using the Delphi method, including a total of 60 indicators such as personal hygiene, disease prevention and control, and diet/nutrition. These indicators can be used to evaluate four aspects of health literacy: health knowledge, healthy behaviors, health beliefs, and health skills. Furthermore, in recent years, scholars have paid increasing attention to discussing the health literacy of media personalities in the HC process [74], the influence of the communication mode on health literacy [75], and the significance of new media in improving public health literacy [76].

Research on Health Information. This research hotspot focused on research concerning the demand for, and acquisition of, health information and the use of the Internet as a source of health information. Scholars found that social media, such as apps, the Internet, WeChat and TikTok, had become the main channels and avenues by which the public obtained health information in China, which, to a large extent, has led to the majority of patients tending to search for relevant health information on the Internet before visiting a doctor [77,78,79]. However, the health information published on the Internet may be incomplete, incorrect, and/or misleading, and Bai [78] therefore suggested that patients and their families should instead seek advice from experts in professional fields, and authoritative medical institutions should provide medical guidelines through social media and the Internet.

Research on AHVM. The study of HC in the AHVM field is in the early stages of exploration in China. Nevertheless, its significance has been recognized by scholars, governments, and enterprises in recent years. In particular, this research hotspot has focused on the application of HC to the practice of AHVM, and scholars have found that HC can effectively enhance the public and workers’ awareness of health management and epidemic prevention and control, decrease the spread of zoonoses, and reduce the risk of contracting foodborne diseases [80,81]. However, the significance and role of HC in the practice of AHVM was to a large extent ignored, due to a lack of expertise in the communication field, resulting in a situation where health knowledge concerning AHVM was not distributed or disseminated effectively. In order to solve this problem, a series of suggestions were made; for example, Gao [82] suggested establishing a professional course on HC at the related AHVM college, while Hu [83] proposed that hospitals and public health organizations should use digital media such as the WeChat public platform, micro-blogs, short videos, and apps for timely reports on the situation in epidemics and other emergencies such as bird flu, to guide public opinion in the correct direction. Excessive reporting and fake information concerning AHVM can produce a significant negative impact [84], and therefore it is important to disseminate the relevant information objectively and impartially.

Research on the Doctor–Patient Relationship. The doctor–patient relationship is changing with the decline of doctors’ patriarchal role and the rise of patients’ autonomous consciousness in China [85]. The media play an important role in building and fostering public understanding of the doctor–patient relationship. As such, this research hotspot focused on the doctor–patient relationship as constructed in media reporting. For example, Gao [86] analyzed reports on the doctor–patient relationship published in the People’s Daily from 1978 to 2018 and found that the status and power over the discourse between doctors and patients were unequal, with doctors being more powerful and having more discursive power. Moreover, positive publicity was still the mainstream view regarding reporting on the doctor–patient relationship, aiming to shape the image of doctors as having good medical ethics, while minimizing the doctor–patient disputes presented in reports [87]. However, in recent years, a special situation has arisen in which social media channels such as Sina Weibo have been keen to hype up or exaggerate doctor–patient conflicts in order to pursue economic interests and cater to the particular psychology of their readers [88]. Therefore, much attention should be paid to profoundly reconsidering and further studying the responsibilities of the media in reporting news relating to the doctor–patient relationship.

Research on Public Health Emergencies. China’s media and academic circles began paying attention to the HC of public health emergencies due to the outbreak of SARS in 2003. At that time, the Chinese Government lacked the capability to respond to such public health emergencies, and the media failed to effectively convey health information related to SARS, resulting in social rumors abounding and thus leading to social chaos [89]. Subsequently, scholars studied the relationship between media reports and crises with regard to China’s HC practices since the SARS epidemic and put forward some suggestions on how the media should better carry out the practice of HC in the face of public health emergencies [90,91,92]. In the last two years, COVID-19 has become an important topic in the HC field in China. Scholars have become committed to research on the communication of information on the risk of COVID-19 in the family [93], the role of We Media and the mainstream media in HC during COVID-19 [94], and the digital generation gaps and health generation gaps that have appeared during the COVID-19 period [95]. A remarkable feature is that the dual role of the government as the authoritative source of information and the main body for HC was emphasized during the COVID-19 period.

#### 5.1.2. The Evolution of Research Hotspots in the Field of HC

**Figure 6:**
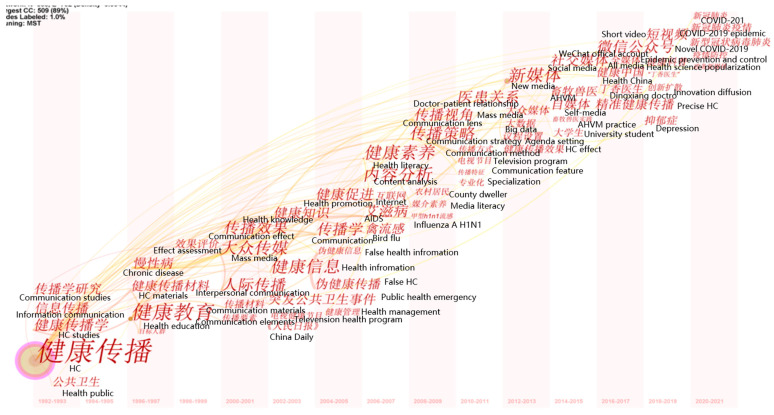
The time-zone map of HC research in China between 1992 and 2021. (Note: the authors added English to the map to aid understanding).

The time-zone map of keywords produced by CiteSpace focused on depicting the evolution of research hotspots chronologically in the field of HC. According to the words with the highest frequency, as shown in Figure 6, this study divided the development of HC research into four stages. Each stage is discussed in combination with the representative articles and key events at that time.

Stage 1 (1992–1999). The highest-frequency keywords that appeared in this stage mainly included HC, communication studies, health education, and information communication (see Figure 6). This stage focused on the study of the basic objectives of HC. The definition of HC was first proposed by Mi G.M. in 1992 and refers to the transmission of credible scientific health information to the public through various communication channels and strategies to promote personal and public health behaviors [96]. Furthermore, it was found that HC was used as a measure for social intervention to improve levels of public health, although this concept was not widely accepted by Chinese academics, even though it established a foundation for follow-up research. Subsequently, scholars in the field of public health discussed the concepts of HC [97,98,99] and laid particular emphasis on the value of communication in the fields of medicine and public health. However, few communication scholars participated in the study of HC during this stage. One remarkable characteristic, however, was that the majority of the academic papers on HC research were published in the *Chinese Journal of Health Education*, founded by the China Health Publicity and Education Association.

Stage 2 (2003–2011). Figure 6 shows the highest-frequency keywords for this period, which mainly included public health emergency, communication knowledge, communication effect, health promotion, health literacy, AIDS, and communication strategy. This indicated that the research themes of HC became more diversified during this period. In addition, the HC on public health emergencies attracted the most attention. One reasonable explanation for this would be the outbreak of SARS in 2003, which led to China experiencing a major public health crisis, bringing about a high level of attention and reflection from the news media, the public, the government, and communication scholars on HC, especially regarding public health, safety, and security. In December of the same year, the “China Health Education and Mass Media Forum” was held in Beijing, making the integration and development of communication and medical and public hygiene a reality for the first time. However, it is worth noting that scholars from the fields of medicine and public health were still the main force driving HC research. In addition, one of the prominent changes was that scholars paid considerable attention to the HC of marginalized groups, e.g., regarding AIDS and homosexuality [100,101]. For example, Yang et al. [90] found that unlicensed prostitutes preferred to learn more about AIDS prevention and treatment from doctors, while drug addicts tended to obtain this knowledge from AIDS prevention and control staff.

Stage 3 (2012–2018). The highest-frequency keywords included new media, mass media, big data, We Media, the WeChat public platform, and All Media (see Figure 6). This indicated that scholars were demonstrating interest in the study of digital media use in HC practices. One possible explanation for this could be that communication scholars gradually became the main force driving HC research, focusing on the role and effects of the application of new media in the HC field. Significantly, scholars held a critical perspective on the role of new media in HC practice. On the one hand, they found that new media could promote the audience’s ability to actively and easily obtain health information, strengthen their health awareness, and construct an equal dialogue and communication between doctors and patients [102,103]. On the other hand, they stressed that new media may cause a series of issues to arise, including the transmission of false health information, excessive health marketing, and the homogenization of communication content [68,104]. Among all the types of new media, great attention was paid to research on the influence of WeChat and apps on audience attitudes and behavior. For example, the forwarding behavior of WeChat users was an important factor in promoting the increase in health information [105].

Stage 4 (2019–2021). In this period, the highest-frequency keywords included short videos, health science popularization, COVID-19, and epidemic prevention and control (see Figure 6). This indicated that scholars focused on the role and influence of short videos during the COVID-19 period. Furthermore, COVID-19 first broke out in China, which caused a great deal of social panic and worry, mainly due to a lack of detailed and accurate information on the situation surrounding the epidemic. Therefore, timely information disclosure and dissemination are particularly critical in public emergencies. In this context, scholars found that short videos play an important role in information communication, media supervision, positive guidance of public opinion, healthy communication, and mobilization of the public [59,65,103]. However, it is necessary to clearly recognize the limitations of short videos, as their ability to warn and reflect on emergencies is relatively weak [106]; they are not the most important means of crisis communication for public emergencies, but rather a supplement and an aid.

### 5.2. Research Frontiers in the Field of HC: Burst Keyword Analysis

**Figure 7:**
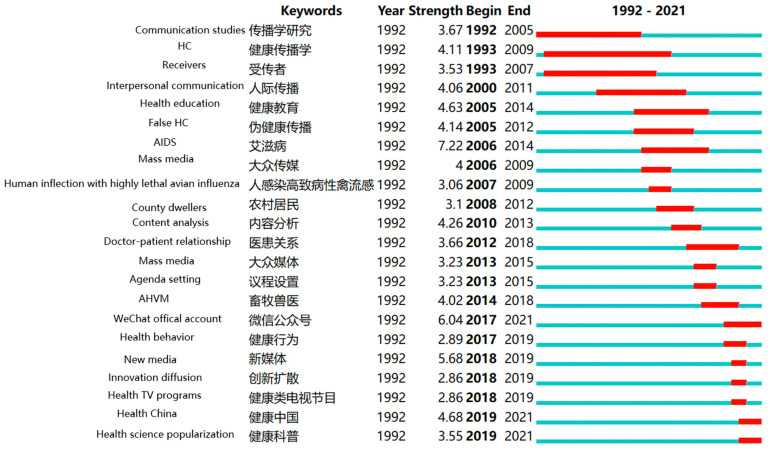
Top 22 keywords with the strongest citation bursts in HC research between 1992–2021. (Note: red shows the period of the detected term burst, while blue presents the time. The authors added English to the map to aid understanding).

A research frontier is defined as an emergent and transient grouping of concepts and underlying research issues [52]. Kleinberg’s [107] burst detection algorithm was adapted to identify emergent research frontier concepts using the burst keyword analysis method, referring to the display of keywords which had rapidly changed in a short period of time or dramatically increased in number, emphasizing abrupt changes in keywords [108]. A burst contains two dimensions: the burst strength and the bursting time. Keywords with high strength can be identified as research frontiers. By considering keywords with high strength, the research frontiers along the timeline for HC research could be roughly determined. After running CiteSpace, the top 22 keywords with high strength in the field of HC were obtained and are listed in Figure 7. From Figure 7, it can be observed that all the strength values were above 2, with the highest at 6.04.

From 1992 to 2002, the keyword with the highest strength was “HC”, which corresponded to that of the first stage in the previous evolution path. During this period, HC was regarded as an emerging discipline, with the majority of the articles on HC being published in the *Chinese Journal of Health Education*. In 1991, the China Institute of Health Education established the Institute of Communication, which specialized in the application of communication to the field of health. As a result, a paper titled “On Health Communication and the Prospect of Health Communication in China” [109] was published in *Journalism Research*, a journal in the field of communication, in 2001. Subsequently, a series of monographs were published with HC as a theme, such as “Heath Communication” [110] and “Principles and Practice of Health Communication” [111]. These academic activities promoted extensive interest in HC studies in the period 1992–2002.

Between 2003 and 2011, the keyword with the highest strength was AIDS, which corresponded to the second stage in the previous evolutionary path. The first case of AIDS in China was discovered in 1985; from then through to the end of the 1990s little attention was paid to AIDS research and practices, as China’s government believed that AIDS would not pose a great challenge to the public health system [101]. It was not until the early 21st century that the government began to attach importance to the major crises caused by AIDS. Specifically, central government officials visited AIDS patients for the first time in 2003 and demonstrated their firm determination to prevent and cure AIDS. This meant that the relationship between the government, the media, and the public had entered a new stage regarding the AIDS issue. In 2006, the Regulation on the Prevention and Treatment of HIV/AIDS was formulated by the State Council, with Article 29 clearly stipulating that “the news media such as broadcast, television, newspapers and the Internet should carry out public welfare propaganda for AIDS prevention and control” [112]. This meant that the mass media should consciously participate in the prevention and treatment of AIDS and should assume the obligation of popularizing and disseminating knowledge on the prevention and treatment of AIDS, to guide public health behaviors and to eliminate social discrimination against HIV-infected persons and HIV patients. In addition, the mass media was the main avenue by which the public obtained AIDS-related information, and thus the media played a vital role in publicizing national AIDS prevention policies and disseminating AIDS information in China. The positive intervention of the media was highly effective in influencing people regarding AIDS. Therefore, the government and media assistance raised extensive concerns about AIDS between 2003 and 2011.

During the period 2012–2018, the keyword with the highest strength was “WeChat official account”, which corresponded to the third stage in the previous evolutionary path. Along with the awakening of the public’s health awareness, HC was no longer solely the responsibility and obligation of government departments and the medical and health system. Individuals, social organizations, and enterprises became involved in the subject of HC, drawing support from various new media to disseminate health information. Among these, the WeChat official account was one of the most popular social network tools and received the most widespread attention in China due to its precision, efficiency, personalization, and interactive qualities [113,114]. The WeChat official accounts are app-based accounts established by individuals or businesses on the WeChat public platform. Through these official accounts, individuals or businesses can communicate and interact with specific groups of words, pictures, and voices in an integrated way, since the WeChat platform is an open platform. At present, WeChat official accounts are widely used by health education and public health institutions. Therefore, WeChat official accounts became a research frontier between 2018 and 2021.

After 2019, “Health China” became the keyword with highest strength, which covered the fourth stage in the previous evolutionary path from a macro perspective. In China, the research and practices regarding HC were initiated by the government from the top down. In fact, the government played the dominant role in the development of HC. Health China is a blueprint for the health of the entire population in an integrated, affluent society, an innovative developmental concept that prioritizes health, and a banner under which the common ideals of the government, society and all people can be united [114]. In essence, it is a political discourse. In fact, it became a research frontier mainly because the central government definitively proposed that it was necessary to “put health into all policies, and ensure health for all” at the meeting of the Political Bureau of the CPC Central Committee in 2016, which showed that the establishment of Health China had been officially elevated to a national strategy. Subsequently, two national-level policies, Outline of Health China 2030 [114] and Opinions of the State Council on Carrying Out the Health China Operation [115], were issued in 2016 and 2019, respectively, stressing that prevention is the most economical and most effective health management strategy, as well as emphasizing adherence to prevention as a priority and advocating a healthy and civilized way of life in order to prevent and control major diseases. Therefore, the issued policies resulted in “Health China” attracting extensive interest in 2019–2021.

## 6. Discussion

It was necessary for this current study to generalize the developmental status and intellectual basis of HC while analyzing the important research issues, providing a significant reference for researchers to obtain a sound understanding of HC research in China and assisting them in mastering the development of science and research issues in the HC field.

In terms of the temporal distribution of publications, increasing attention has been directed toward HC research over time from 1992 to 2021 in China. The first article, titled “On Communication and Health Communication” [96] was published in the *Chinese Journal of Health Education* in 1992. In the initial stage, a remarkable feature is that scholars in the fields of medicine and public health were the main research drivers for HC, while communication scholars were absent. To a large extent, HC was regarded as part of the medical and public health discourse during this stage. Subsequently, due to the outbreak of severe acute respiratory syndrome (SARS), communication scholars were driven to devote attention to applying communication theory to interpret this public health emergency. This revealed that HC had begun to present an interdisciplinary discourse involving communication, public health, and medicine. Meanwhile, the content of HC was changing from “providing biomedical knowledge” to “promoting behavior change” [R]. Throughout the overall development of HC in China, the main force driving HC research changed from medical and public health scholars to communication scholars. One possible explanation is that social media became an important channel for HC [68].

Scientific cooperation is a process in which two or more authors share their resources and talents and create studies together [37]. A sound collaborative relationship among scholars can establish academic networks in order to share innovative ideas, concepts, and theories, generate new knowledge, and ultimately reduce the waste of academic resources and improve the productivity of research [50]. However, in the HC field, the cooperation between highly productive scholars is relatively weak. These researchers have a wide range of research interests, mainly including effectiveness evaluation of HC, public health emergency management, social media application in HC, and health promotion [53,54,55]. Nevertheless, cooperative relationships among scholars do exist on a small scale, usually occurring between colleagues. In terms of research institutions, strong partnerships among the high-yield authors have not yet been formed in the HC field. Universities are the leading force in HC research in China, representing the research directions and core issues of HC. The top four universities in the field are well-known universities in China. One possible explanation is that these universities may invest more manpower and more financial and material resources into the implementation of HC research in China.

In terms of research hotspots, scholars have shown great interest in health education, new media, health literacy, health information, AHVM, doctor–patient relationships, and public health emergencies. Moreover, the relationship between social media and COVID-19 has been extensively discussed. The evolutionary trends regarding HC have gone through four stages. First, there was an early stage (1992–2002), where research was primarily focused on the discussions surrounding the conception of HC, mainly from the medical and public health perspectives, involving the topic of health education and communication studies. In the second stage (2003–2011), HC research began to diversify, eventually involving public health emergencies, the effects of communication, AIDS, the promotion of good health, and communications strategies, with public health emergencies and AIDS attracting the most attention. In the third stage (2012–2018), research shifted focus towards the application of new media in HC research and practice. This reflected the fact that the status of these media in the HC field was increasingly valued. In the fourth stage (2019–2021), a significant body of practical and theoretical explorations were conducted with the aim of providing the public with objective and correct information concerning COVID-19, in order to relieve panic and depression. In summary, HC research in China has continuously become diversified and more in-depth.

In terms of the research frontiers, from 1992 to 2002, HC as an emerging discipline was the research frontier. Academic activities were promoted by scholars and practitioners during this period. In the following period (2003–2011), AIDS became the research frontier due to the government’s support and reporting by the media. Between 2012 and 2018, the research frontier was WeChat official accounts due to the convenience of the platform and the public’s greater awareness of health concerns [114]. After 2019, Health China became the research frontier due to the policies that were implemented regarding HC.

In general, the visualized knowledge maps produced by CiteSpace offer a profound understanding of the major authors and institutions, research hotspots, evolutionary progress, and research frontiers in the field of HC in China. The results mentioned above can thus provide valuable information to HC researchers as well as practitioners. For example, this study can offer HC practitioners accurate information concerning the major authors and institutions in order to assist them in drafting and enacting appropriate HC rules/policies. In addition, unlike previous studies, this study reviewed HC research as a whole rather than emphasizing or focusing specifically on one of its subfields. Moreover, we employed a bibliometric analysis to objectively review the HC literature, which was a supplement to the early qualitative HC review.

## 7. Conclusions and Future Research

To the best of our knowledge, this is the first study to employ a bibliometric method to analyze the corpus of Chinese HC literature over the past three decades systematically and comprehensively. As such, this study aimed to: (1) discuss the scientific achievements in order to explore the basic characteristics and the current status and development of HC research in China; (2) identify the contributions of the core authors and institutions by analyzing the co-authorship collaboration network map; (3) explore the important research topics, evolutionary paths and research frontiers by analyzing a co-word network map. The main findings were as follows:

(1) In China, the number of HC publications has exhibited a noticeable upward trend over time between 1992 and 2021. Three developmental stages can be identified: a slow development stage (1992–2004), a steady development stage (2005–2015), and a rapid development stage (2016–2021). In addition, although HC represents a transdisciplinary field, the top journals in the communication field have played a leading role in the study of HC in recent years.

(2) The most productive authors in this field included Wang L.Y., Zhang, Z.L., Wang Y.L., Wang, L., and Hong, T. However, a strong partnership among productive authors has not yet been formed. The high-yield research institutions mainly included the Communication University of China, Renmin University, Fudan University, the China Health Education Center, and Peking University. Collaboration between productive institutions is weak.

(3) In terms of research hotspots, seven research hotspots were identified as follows: health education, new media, health literacy, health information, AHVM, the doctor–patient relationship and public health emergencies. The development of HC research was divided into four stages. The first stage (1992–1999) focused on the study of the basic objectives of HC. In the second stage (2003–2011), the research themes of HC became more diversified. The third stage (2012–2018) focused on the study of digital media use in HC practices. In the fourth stage, the focus of research was the role and influence of short videos during the COVID-19 period. In terms of research frontiers, four main research frontiers were identified in different periods: HC, AIDS, WeChat official accounts and Health China.

However, this study has several limitations. For example, we conducted this study in a Chinese context, including collecting the data from a Chinese scientific database, which unfortunately fails to provide readers with data on HC research worldwide. We believe that we selected the right keywords to answer the research questions; however, it will be necessary to amend the keywords in the future to retrieve other documents more accurately.

Considering the above-mentioned conclusions, we propose several topics for future research. First, we propose research on the influence of social media on the behavior, attitudes, and knowledge of users in the context of HC. Second, we propose research on the new situations and the characteristics and concepts of HC in the post COVID-19 era. Third, we recommend analysis of the digital generation gap and the health generation gap in China. Fourth, we propose the analysis of the influence of big data, artificial intelligence, and cloud-based medical treatment on HC. Last but not least, a comparative analysis of different national databases with regard to HC could be conducted, in order to obtain a deeper understanding of the state of HC research worldwide.

## Figures and Tables

**Table 1:** Top 10 journals ranked by the number of publications in the field of HC.

Journal Names	Quantity
Chinese Journal of Journalism and Communication	30
Modern Communication (Journal of Communication University of China)	28
Journalism and Communication	25
Journal of Southwest Minzu University (Humanities and Social Science)	12
Chinese Medical Ethics	11
Chinese Journal of Medical Library and Information Science	10
Acta Editologica	5
Chinese Journal of School Health	3
Chinese Journal of Public Health	3
Journal of Zhejiang University (Humanities and Social Sciences)	3

**Table 2:** The most productive authors in the field of HC in China.

No.	Author	Frequency	Proportion	Year
1	Wang, L.Y.	9	0.60%	2016
2	Zhang, Z.L.	6	0.40%	2001
3	Wang, Y.L.	6	0.40%	2015
4	Wang, L.	6	0.40%	2013
5	Hong, T	6	0.40%	1999
6	Yu, G.H.	6	0.40%	2011
7	Wei, N.F.	6	0.40%	2003
8	Yu, Y.H.	6	0.40%	2013
9	Mi, G.M.	5	0.33%	1992
10	Zhu, B.	5	0.33%	2006
11	Tian, X.Y.	5	0.33%	2009
12	Niu, W.Y.	5	0.33%	2004
13	Jiang, H.	5	0.33%	2018
14	Hu, B.J.	5	0.33%	2012
15	Fu, H.	5	0.33%	2013

Note: frequency refers to the number of papers published.

**Table 3:** Top 10 research institutions in the field of HC in China.

Rank	Institution	Frequency	Proportion	Year
1	Communication University of China	80	5.32%	2005
2	Renmin University of China	39	2.59%	2010
3	Fudan University	30	1.99%	2001
4	China Health Education Center	28	1.86%	2009
5	Peking University	26	1.72%	2004
6	Shanghai Jiao Tong University	20	1.32%	2009
7	Chinese Center for Disease Control and Prevention	20	1.32%	2005
8	Nanchang University	17	1.13%	2011
9	Shanghai Institute of Health Education	15	1.00%	2009
10	Nanjing University	12	0.80%	2008

**Table 4:** Top 10 keywords in the field of HC in China.

Rank	Keyword	Frequency	BC	Year
1	HC	937	1.25	1992
2	Health education	105	0.16	1996
3	New media	93	0.13	2012
4	Health literacy	47	0.03	2007
5	WeChat public platform	39	0.03	2016
6	COVID-19	39	/	2020
7	AHVM	38	/	2014
8	Health information	36	0.07	2003
9	Social media	28	0.05	2014
10	Doctor–patient relationship	28	0.04	2009
11	Public health emergency	26	0.03	2003

## Abbreviations

AHVM	Animal husbandry and veterinary medicine
AIDS	Acquired immunodeficiency syndrome
BC	Betweenness centrality
COVID-19	Coronavirus disease
CNKI	China National Knowledge Infrastructure
HC	Health communication
HIV	Human immunodeficiency virus
H7N9	Asian lineage avian influenza A virus
SARS	Severe acute respiratory syndrome
